# Integrated Multidisciplinary Approach to Advance Care Planning for Vulnerable Older Adults

**DOI:** 10.1093/geroni/igaa057.788

**Published:** 2020-12-16

**Authors:** Jennifer Gabbard, Nicholas Pajewski, Kathryn Callahan, Ajay Dharod, Kristie Foley, Jeff Williamson

**Affiliations:** Wake Forest School of Medicine, Winston-Salem, North Carolina, United States

## Abstract

Advance Care Planning (ACP) is increasingly recognized as a crucial step to ensure patients receive medical care that consistent with their overall goals and values; however it remains suboptimal among vulnerable older adults. The objective was to determine whether a nurse navigator led ACP pathway plus a provider-facing EMR documentation program called ACPSmart improves ACP documentation within the EMR within an Accountable Care Organization (ACO) for vulnerable older adults. This was a randomized, pragmatic, effectiveness clinical trial was conducted from November 1, 2018 to November 1, 2019, at 8 primary care practices. Patients 65 years or older within an affiliated ACO with multimorbidity plus physical impairments, cognitive impairments, and/or frailty were included. Participants were randomized to either a nurse navigator led ACP pathway (NN) or usual care (UC). The primary outcome was documentation of new ACP discussion within the EMR at 12 months along with the quality of ACP discussions. Among 759 randomized patients (379 NN / 380 UC, mean age 77.7 years), compared to usual care, the NN led ACP pathway resulted in a higher rate of ACP documentation (42.2% vs 3.7%, p<0.001). There was also higher completion rates of ACP legal forms (24.3% vs 10.0%, p<0.001), MOST forms (10.3% vs 1.1%, p<0.001), along with documentation of medical decision maker (64% vs 35%, p<0.001). The nurse navigator led ACP pathway plus ACPSmart documentation program increased documentation of ACP within the EMR. This may mitigate prior barriers to ACP and could substantially improve ACP documentation within the EMR.

